# Sex-based epidemiological and immunovirological characteristics of people living with HIV in current follow-up at a tertiary hospital: a comparative retrospective study, Catalonia, Spain, 1982 to 2020

**DOI:** 10.2807/1560-7917.ES.2023.28.10.2200317

**Published:** 2023-03-09

**Authors:** Sara Toyos, Leire Berrocal, Ana González-Cordón, Alexy Inciarte, Lorena de la Mora, María Martínez-Rebollar, Montserrat Laguno, Emma Fernández, Juan Ambrosioni, Iván Chivite, Elisa de Lazzari, José Luis Blanco, Esteban Martínez, José M Miró, Josep Mallolas, Berta Torres

**Affiliations:** 1 Hospital Verge de la Cinta, Tortosa, University of Barcelona, Barcelona, Spain; 2 HIV Unit, Infectious Diseases Service, Hospital Clínic, Barcelona, Spain, Institut d’Investigacions Biomèdiques August Pi i Sunyer (IDIBAPS), University of Barcelona, Barcelona, Spain; 3 CIBER de Enfermedades Infecciosas (CIBERINFEC), Instituto de Salud Carlos III, Madrid, Spain

**Keywords:** HIV, women, sex differences, cohort study, epidemiology

## Abstract

**Background:**

Epidemiological and immunovirological features of people living with HIV (PLWH) can vary by sex.

**Aim:**

To investigate, particularly according to sex, characteristics of PLWH who consulted a tertiary hospital in Barcelona, Spain, in 1982–2020.

**Methods:**

PLWH, still in active follow-up in 2020 were retrospectively analysed by sex, age at diagnosis, age at data extraction (December 2020), birth place, CD4^+^ cell counts, and virological failure.

**Results:**

In total, 5,377 PLWH (comprising 828 women; 15%) were included. HIV diagnoses in women appeared to decrease from the 1990s, representing 7.4% (61/828) of new diagnoses in 2015–2020. From 1997, proportions of new HIV diagnoses from patients born in Latin America seemed to increase; moreover, for women born outside of Spain, the median age at diagnosis appeared to become younger than for those born in Spain, with significant differences observed in 2005–2009 and 2010–2014 (31 vs 39 years (p = 0.001), and 32 vs 42 years (p < 0.001) respectively), but not in 2015–2020 (35 vs 42 years; p = 0.254). Among women, proportions of late diagnoses (CD4^+^ cells/mm^3^ < 350) were higher than men (significantly in 2015–2020: 62% (32/52) vs 46% (300/656); p = 0.030). Initially, virological failure rates were higher in women than men, but they were similar in 2015–2020 (12% (6/52) vs 8% (55/659); p = 0.431). Women ≥ 50 years old represented 68% (564/828) of women actively followed up in 2020.

**Conclusions:**

Women still have higher rates of late HIV diagnoses than men. Among currently-followed-up women, ≥ 50 year-olds, who need age-adapted care represent a high percentage. Stratifying PLWH by sex matters for HIV prevention and control interventions.

**Key public health message**

**What did you want to address in this study?**

We wanted to find out how numbers of HIV diagnoses and patient features have evolved since the beginning of the HIV epidemic between 1982 and 2020. We looked at demographic and clinical characteristics of people living with HIV who consulted a hospital in Barcelona, Spain, and analysed their characteristics, particularly according to their sex assigned at birth. Our focus is on women living with HIV, their age, country of origin, the timeliness of their diagnosis and treatment.

**What have we learnt from this study?**

Numbers of HIV diagnoses in women in our centre decreased in the last decade. Among women, the proportions originating from Latin America increased progressively, representing 43% of new HIV diagnoses in 2015–2020. Proportions of women diagnosed late among women living with HIV were higher than the same type of proportions among men. Women older than 50 years who would need adapted care, represented a high percentage of patients currently being followed up.

**What are the implications of your findings for public health?**

Women living with HIV are still underrepresented in the scientific literature in terms of their demographic, epidemiological, pharmacological, and long-term health characteristics. Determining these characteristics in different settings and contexts is especially important for adequate strategies to prevent and control HIV.

## Introduction

Worldwide, women and girls in 2021 represented ca 54% of people living with HIV (PLWH) and 49% of all new HIV diagnoses were in women. Across different parts of the globe, numbers of women living with HIV (WLWH) and their age at diagnosis can vary. In sub-Saharan Africa, 15 to 24-year-old women are estimated twice as likely to be living with HIV as men of the same age. In all other areas of the world, men living with HIV (MLWH) outnumber WLWH [1].

Studying WLWH and MLWH separately is important for several reasons. In terms of pathogenesis, issues related to both gender and sex may cause differences between women and men. Gender-specific behaviours and or/socio-cultural constructs could influence how HIV is acquired in each group or how it affects respective individuals. Sex-related biological aspects, such as genetic and/or hormonal factors may also lead to heterogeneous host-responses to the virus in men and women, with, for example, variable levels of immune activation at acquisition of the virus/onset of disease and differences in tolerances to antiretroviral medications [2]. In terms of preventive strategies, WLWH may receive less attention and may be excluded in countries where the epidemic disproportionally affects men who have sex with men [3].

According to the Spanish Bulletin of Epidemiological Surveillance of HIV and AIDS published in 2022, the rate of new HIV diagnoses in Spain is similar to that of countries in the western part of the World Health Organization (WHO) European Region. In 2021, women accounted for 11% of all new HIV diagnoses in Spain, with a rate of 1.3 new HIV diagnoses per 100,000 population [4], which is slightly lower than the 1.6 new HIV diagnoses per 100,000 population reported among women across the countries of the European Economic Area in 2021 [5].

Concerning the region of Catalonia (7.5 million inhabitants), the Hospital Clínic in Barcelona, a tertiary hospital, has been one of the reference centres for HIV care since the beginning of the HIV epidemic. In 2020, 329 cases of HIV were diagnosed in Catalonia [6], and in the same year 110 treatment-naïve HIV patients had their first visit at the Hospital Clínic (according to local hospital data).

The objective of this study was to investigate epidemiological and immunovirological characteristics of PLWH at diagnosis, at Hospital Clínic, and the response of these patients to antiretroviral treatment (ART). The aim was also to understand how these features differ among PLWH, in particular according to sex, and how they evolved with time since the beginning of the HIV epidemic.

## Methods

### Study type

This was a descriptive, retrospective and comparative observational study of PLWH who were followed-up in the HIV unit of Hospital Clínic from the beginning of the HIV epidemic until December 2020, when data extraction was performed.

### Source of data

Epidemiological data, such as date and place of birth, and date of HIV diagnosis, have been routinely registered into a clinical-history database of Hospital Clínic approved by the local ethics review committee since 1982. Laboratory data, such as CD4^+^ cell count, HIV viral load, and type of ART, have been routinely registered in the same database since 1990.

### Description of the historical and active cohorts and their use

The ‘historical cohort’ of Hospital Clínic includes all PLWH who visited Hospital Clínic since the HIV epidemic started. The ‘active cohort’ of Hospital Clínic consists of PLWH who are currently in follow-up, i.e. HIV patients who had at least one laboratory test 12 months before the data extraction was performed (December 2020).

The historical cohort was used to describe all patients with a new HIV diagnosis who ever visited our hospital. These were described according to each year of the study and stratified according to sex.

The active cohort served as a base for more extensive epidemiological, immunovirological and clinical analyses. Data in the active cohort were stratified by sex and retrospectively analysed according to successive periods within the study. The time intervals of these periods (all starting on 1 January and ending on 31 December of the given years) were determined by considering the introduction of combined ART, commercialisation of newer drugs, and changes in policies on when to start ART. Two periods pre-introduction of combined ART and four periods post-introduction of combined ART were established: period 1, from January 1982 to December 1989; period 2, from January 1990 to December 1996; period 3, from January 1997 to December 2004; period 4, from January 2005 to December 2009; period 5, from January 2010 to December 2014; and period 6, from January 2015 to December 2020.

### Epidemiological variables and laboratory parameters of active cohort patients

For the active cohort, the epidemiological variables studied were sex (referring to, throughout the manuscript, as ‘sex assigned at birth’; male or female), year of HIV diagnosis, age at HIV diagnosis, age at data extraction (December 2020), mode of HIV acquisition (sexual, transfusion of blood products, vertical transmission, or people who inject drugs) and place of birth (Spain, rest of Europe, Africa, Latin America, United States, Asia, or unknown).

Laboratory, antiretroviral and clinical data were only analysed for a subset of the active cohort starting 1990. Indeed, in patients diagnosed with HIV before 1990, missing data on the baseline CD4^+^ cell count, HIV viral load, and antiretroviral treatment (ART) were frequent occurrences; hence, the period from 1 January 1982 to December 1989 was excluded from analysis. Moreover, for patients transferred to Hospital Clínic from other centres, the baseline CD4^+^ cell count and HIV viral load were not always reported in the electronic records; therefore, for data accuracy, laboratory, antiretroviral and clinical data were analysed only for treatment-naïve HIV patients visiting in our hospital, i.e. patients who had never started ART in other centres before being transferred or before their first visit to our hospital.

The laboratory parameters taken into account were the CD4^+^ cell count (cells/mm^3^) at diagnosis, nadir CD4^+^ cell count (cells/mm^3^), and HIV viral load (copies/mL) at diagnosis. Late diagnosis was defined as CD4^+^ cell count  < 350 cells/mm^3^ at diagnosis. In relation to treatment, we analysed the type of ART regimen at the initiation of treatment, number of changes in ART during the follow-up, and virological suppression and virological failure after the initiation of ART. Virological failure was defined as two consecutive viral loads of > 50 copies/mL after achieving viral suppression.

### Statistical analyses

Qualitative variables were expressed as the frequency and percentage. Quantitative variables were expressed as the median and interquartile range (IQR), as some of them were not normally distributed. Dunn’s test was used to perform multiple pairwise comparisons. For comparisons of two groups in different time periods, logistic and linear regression models with the interaction of both group and period variables were performed for each variable of interest as the dependent variable. Application criteria for both regressions were checked, including the normal distribution of the residuals for linear regression. All tests were two-tailed, and statistical significance was set at p < 0.05. The statistical analyses were performed using Stata 17 software (StataCorp LLC, College Station, TX, United States (US)).

## Results

### Demographic evolution of the historical cohort

**Figure 1:**
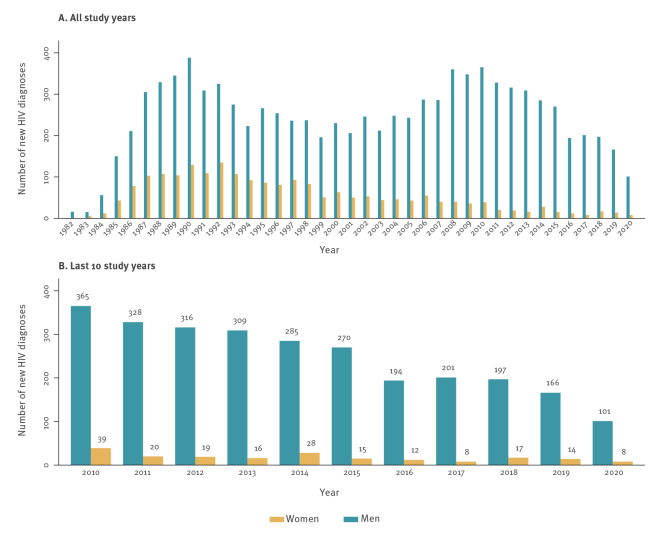
Number of new HIV diagnoses per year and by sex at Hospital Clínic (A) over the whole study time (n = 11,617) and (B) in the last 10 years of the study (n = 2,926), Barcelona, Spain, 1982–2020

From the first cases registered in the early 1980s to those registered until December 2020, 11,617 PLWH were followed-up in the Hospital Clínic. Women accounted for 18% (n = 2,084) of all PLWH during this period. The number of female patients with a new HIV diagnosis who ever visited Hospital Clínic appeared to increase in the late 1980s, then to progressively decrease after 1992 **(** **Figure 1** **).**

For the panel B, the exact number of new HIV diagnoses figure above each chart bar.

### Epidemiological characteristics of the active cohort

Only PLWH who underwent active follow-up until December 2020, when the analysis was performed (n = 5,377), were included in the more detailed study of demographic and epidemiological characteristics. Women represented 15% (828/5,377) of the active cohort with a median follow-up of 18 years (IQR: 10.5–23.0 years). As observed in the historical cohort, a progressive decrease in the number of HIV diagnoses in women was observed since the 1990s. New diagnoses in women represented 33.1% (274/828) of the total diagnoses in 1990–1996 vs 7.4% (61/828) in 2015–2020.

The analysis of epidemiological data was performed in all women (n = 828) and men (n = 4,549) in the active cohort. Most of the women who were currently in follow-up at Hospital Clínic (73.1%, 605/828) were diagnosed with HIV between the ages of 20 and 39 years and an age at diagnosis of ≥ 50 years was observed in 7.4% (61/828) of the women, with no overall differences with the men (75.5%, 3,434/4,549 and 6.2%, 282/4,549, respectively). Women were younger at diagnosis than men, when diagnosed before 1997, but this trend reversed with time, and women appeared significantly older than men at diagnosis (36 vs 32 years old; p = 0.005) in the last study period from 2015 to 2020 **(** **Table 1** **)**.

**Table 1:** Comparison of age, basal laboratory data, late diagnosis and virological failure between women living with HIV and men living with HIV in Barcelona, Spain, 1982–2020 (n = 5,377)

Comparison of women and men living with HIV										
**Women (n = 828)^a^ and men’s (n = 4,549)^a^ age at diagnosis, 1982–2020**										
Periods	Age in years at diagnosis							Regression coefficient	95% CI	p value
	Women n = 828			Men n = 4,549						
	N	Median (IQR)		N		Median (IQR)				
1982–1989	96	23 (20 to 26)		215		25 (22 to 29)		−2.45	−4.68 to −0.22	0.031
1990–1996	274	27 (24 to 32)		536		30 (26 to 36)		−3.63	−4.98 to −2.28	< 0.001
1997–2004	222	33 (28 to 41)		842		33 (28 to 39)		0.33	−1.04 to 1.70	0.636
2005–2009	100	35 (29 to 43)		893		33 (28 to 40)		1.82	−0.10 to 3.73	0.063
2010–2014	75	34 (27 to 43)		1112		33 (27 to 40)		1.78	−0.39 to 3.94	0.109
2015–2020	61	36 (30 to 44)		951		32 (27 to 39)		3.44	1.04 to 5.84	0.005
TOTAL	828	30 (25 to 37)		4,549		32 (27 to 39)		−1.95	−2.66 to −1.23	<0.001
**Laboratory parameters for women (n = 549)^b^ and men (n = 2,787)^b^, 1990–2020**										
Periods	CD4^+^ cells/mm^3^ ‒ basal							Comparison		
	Women n = 540^c^			Men n = 2,737^c^				Regression coefficient	95% CI	p value
	N	Median (IQR)		N		Median (IQR)				
1990‒1996	176	292 (200 to 407)		136		347 (171 to 540)		−49.05	−105.58 to 7.49	0.089
1997‒2004	176	291 (151 to 492)		532		302 (134 to 495)		4.08	−38.98 to 47.14	0.853
2005‒2009	80	233 (110 to 495)		635		383 (235 to 550)		−67.87	−126.61 to −9.12	**0.024**
2010‒2014	56	381 (207 to 482)		778		376 (242 to 509)		−6.38	−74.89 to 62.14	0.855
2015‒2020	52	313 (153 to 445)		656		379 (219 to 550)		−72.11	−143.45 to −0.77	**0.048**
TOTAL	540	289 (176 to 457)		2,737		365 (213 to 528)		−42.92	−66.32 to −19.52	<0.001
Periods	CD4^+^ cells/mm^3^ ‒ nadir							Regression coefficient	95% CI	p value
	Women n = 548^c^			Men n = 2,786^c^						
	N	Median (IQR)		N		Median (IQR)				
1990‒1996	184	227 (131 to 291)		152		225 (130 to 332)		−13.30	−50.47 to 23.87	0.483
1997‒2004	177	207 (92 to 293)		554		208 (99 to 311)		−11.67	−40.95 to 17.61	0.435
2005‒2009	80	201 (98 to 295)		641		288 (198 to 380)		−56.28	−96.49 to −16.07	**0.006**
2010‒2014	56	304 (189 to 379)		781		326 (208 to 439)		−32.35	−79.27 to 14.56	0.176
2015‒2020	51	269 (157 to 433)		658		355 (209 to 497)		−63.44	−112.73 to −14.14	**0.012**
TOTAL	548	225 (114 to 310)		2,786		289 (169 to 409)		−68.04	−84.60 to −51.47	<0.001
Periods	Log HIV viral load ‒ basal							Regression coefficient	95% CI	p value
	Women n = 424^c^			Men n = 2,755^c^						
	N	Median (IQR)		N		Median (IQR)				
1990‒1996	78	4.29 (3.19 to 4.82)		139		4.81 (4.18 to 5.26)		−0.65	−0.91 to −0.39	**< 0.001**
1997‒2004	163	4.83 (4.21 to 5.42)		544		5.10 (4.55 to 5.51)		−0.30	−0.46 to −0.14	**< 0.001**
2005‒2009	77	4.36 (3.82 to 5.03)		637		4.69 (4.15 to 5.17)		−0.22	−0.44 to 0.01	0.055
2010‒2014	55	4.19 (3.47 to 4.94)		779		4.56 (4.02 to 5.1)		−0.44	−0.69 to −0.18	**0.001**
2015‒2020	51	4.31 (3.53 to 5.03)		656		4.66 (3.94 to 5.31)		−0.32	−0.59 to −0.06	**0.017**
TOTAL	424	4.47 (3.77 to 5.15)		2,755		4.73 (4.13 to 5.28)		−0.27	−0.36 to −0.17	**< 0.001**
**Proportion of individuals with late diagnosis and virological failure among women (n = 549)^b^ and men (n = 2,787)^b^, 1990–2020**										
Periods	Late diagnosis							Odds ratio	95% CI	p value
	Women (total = 540)^c^			Men (total = 2,737)^c^						
	n	N	%	n	N		%			
1990‒1996	109	176	62	68	136		50	1.63	1.03 to 2.56	**0.035**
1997‒2004	102	176	58	303	532		57	1.04	0.74 to 1.47	0.816
2005‒2009	52	80	65	280	635		44	2.36	1.45 to 3.83	**0.001**
2010‒2014	25	56	45	350	778		45	0.99	0.57 to 1.70	0.960
2015‒2020	32	52	62	300	656		46	1.90	1.06 to 3.39	**0.030**
TOTAL	320	540	60	1,301	2,737		48	1.61	1.33 to 1.94	**< 0.001**
Periods	Virological failure							Odds ratio	95% CI	p value
	Women (total = 549)^c^			Men (total = 2,787)^c^						
	n	N	%	n	N		%			
1990‒1996	137	184	74	104	152		68	1.35	0.84 to 2.17	0.222
1997‒2004	95	177	54	265	554		48	1.26	0.90 to 1.77	0.177
2005‒2009	30	80	38	163	641		25	1.76	1.08 to 2.86	**0.023**
2010‒2014	13	56	23	102	781		13	2.01	1.05 to 3.87	**0.036**
2015‒2020	6	52	12	55	659		8	1.43	0.59 to 3.50	0.431
TOTAL	281	549	51	689	2,787		25	3.19	2.65 to 3.85	**< 0.001**

CI: confidence interval; IQR: interquartile range; NA: not applicable.

^a^ Age was evaluated in all patients of the active cohort.

^b^ Laboratory parameters were evaluated in only treatment-naïve HIV patients of the active cohort, who were diagnosed with HIV after 1990.

^c^ Numbers presented concern individuals among the 3,336 between 1990 and 2020 (549 women and 2,797 men), who had data available for the parameters in question.

Quantitative data are reported as the median (IQR). Dichotomous variables (late diagnosis and virological failure) are expressed as the frequency of patients presenting with the characteristic in relation to the total number of patients for that period with available data (percentage). Regression models with the interaction of both the group and period variables were performed for each variable of interest as the dependent variable. For quantitative data, linear regression was performed and the regression coefficient of the model was reported. For dichotomous variables, logistic regression was performed and the odds ratio of the model was reported. Both 95% confidence intervals and p values were reported for all models.

**Figure 2:**
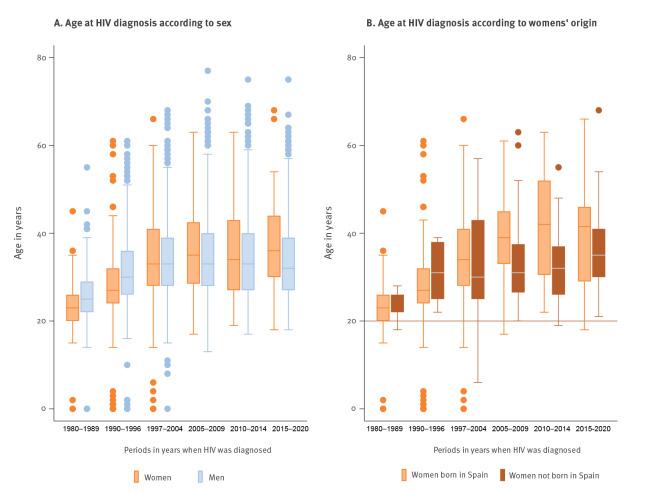
Distribution of median age at diagnosis of HIV over time, stratified by (A) sex (n = 5,377) and, for women only, (B) origin (n = 820)^a^, Barcelona, Spain, 1982–2020

Among women, younger age at diagnosis was observed for those diagnosed before 1997 compared to women diagnosed later. In the first two periods (spanning 1982 to 1996), the median ages at diagnosis were 23 and 27 years, while in the four subsequent periods these were 33, 35, 34, and 36 years (p < 0.001 for all periods). When we grouped age at diagnosis by place of birth, women born in a foreign country with an HIV diagnosis after 2005 were younger than women born in Spain, and this difference was significant in both the 2005–2009 (31 vs 39 years old; p = 0.001) and 2010–2014 (32 vs 42 years old; p < 0.001) periods **(** **Table 2** and **Figure 2** **). **Also, for all new diagnoses among women under 40 years in 2015–2020, almost half of them (49%, 18/37) were born in Latin America, in contrast with 27% (10/37) of women born in Spain for the same period.

**Table 2:** Comparison of age, late diagnosis, and virological failure between Spanish and foreign-born women living with HIV in Barcelona, Spain, 1982‒2020 (n = 820)^a^

Comparison of Spanish and foreign-born women living with HIV										
**Age at diagnosis of women according to country of birth, 1982 to 2020 (n = 820)^b^**										
Periods	Age in years at diagnosis							Regression coefficient	95% CI	p value
	Born in Spain n = 629			Foreign−born n = 191						
	N	Median (IQR)		N		Median (IQR)				
1982–1989	91	23 (20 to 26)		5		22 (22 to 26)		−0.10	−8.98 to 8.78	0.982
1990–1996	260	27 (24 to 32)		10		31 (25 to 38)		−3.77	−10.01 to 2.46	0.235
1997–2004	176	34 (28 to 41)		43		30 (25 to 43)		1.92	−1.37 to 5.21	0.253
2005–2009	52	39 (33 to 45)		48		31 (27 to 38)		6.57	2.70 to 10.44	0.001
2010–2014	28	42 (31 to 52)		47		32 (26 to 37)		9.75	5.13 to 14.37	< 0.001
2015–2020	22	42 (29 to 46)		38		35 (30 to 41)		3.01	−2.17 to 8.20	0.254
TOTAL	629	29 (24 to 36)		191		31 (26 to 38)		−2.08	−3.87 to −0.29	0.023
**Late diagnosis and virological failure among Spanish (n = 432)^c^ and foreign-born (n = 129)^c^, 1990 to 2020**										
Periods	Late diagnosis							Odds ratio	95% CI	p value
	Born in Spain (total = 404)^d^			Foreign born (total = 128)^d^						
	n	N	%	n	N		%			
1990–1996	103	168	61	3	4		75	0.53	0.05 to 5.19	0.584
1997–2004	86	143	60	15	30		50	1.51	0.68 to 3.32	0.308
2005–2009	27	46	59	25	34		74	0.51	0.20 to 1.34	0.172
2010–2014	13	26	50	12	30		40	1.50	0.52 to 4.33	0.454
2015–2020	11	21	52	21	30		70	0.47	0.15 to 1.50	0.203
TOTAL	240	404	59	76	128		59	1.01	0.67 to 1.50	0.995
Periods	Virological failure							Odds ratio	95% CI	p value
	Born in Spain (total = 412)^d^			Foreign born (total = 129)^d^						
	n	N	%	n	N		%			
1990–1996	130	175	74	4	5		80	0.72	0.08 to 6.63	0.774
1997–2004	77	144	53	17	30		57	0.88	0.40 to 1.94	0.750
2005–2009	17	46	37	13	34		38	0.95	0.38 to 2.36	0.907
2010–2014	6	26	23	7	30		23	0.99	0.28 to 3.42	0.982
2015–2020	3	21	14	3	30		10	1.50	0.27 to 8.28	0.642
TOTAL	233	412	57	44	129		35	2.51	1.66 to 3.80	< 0.001

CI: confidence interval; IQR: interquartile range; NA: not applicable.

^a^ Information on place of birth was missing for eight women.

^b^ Age was evaluated in all 820 women with data on place of birth.

^c^ Laboratory parameters were evaluated in only treatment-naïve HIV patients of the active cohort who were diagnosed with HIV after 1990.

^d^ Numbers are for individuals who had data available for the parameters in question.

Quantitative data are reported as the median (IQR). Dichotomous variables (late diagnosis and virological failure) are reported as the frequency of patients presenting the characteristic in relation to the total number of patients for that period with available data (percentage). Regression models with the interaction terms for both the group and period variables were performed for each variable of interest as the dependent variable. For quantitative data, linear regression was performed and the regression coefficient of the model was reported. For dichotomous variables, logistic regression was performed and the odds ratio of the model was reported. Both 95% confidence intervals and p values were reported for all models.

IQR: interquartile range.

^a^ Origin was characterised as place of birth in Spain or elsewhere. Information on place of birth was missing for eight women.

Box lines represent the 25^th^ (Q1), 50^th^ (median), and 75^th^ (Q3) percentiles. Adjacent lines represent the lower and upper adjacent values (i.e. lowest and highest observations within the range defined as follows: Q1 − 1.5 × IQR for the lower limit; Q3 − 1.5 × IQR for the upper limit). Dots represent outliers.

In panel B, for women not born in Spain, the line for Q1 overlaps with the line for the median.

**Figure 3:**
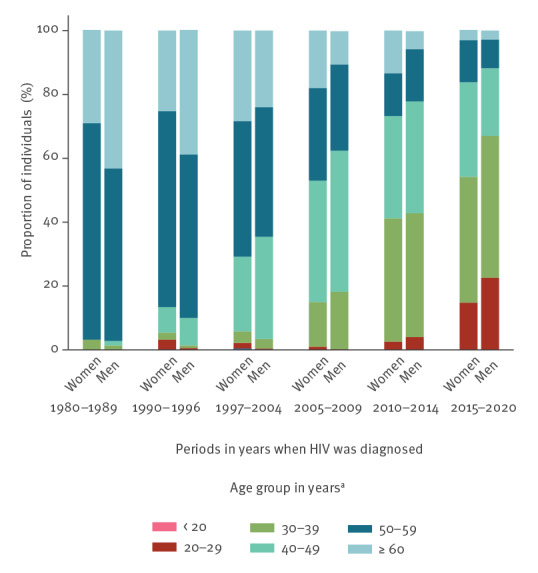
Age groups of women and men^a^ in active follow-up stratified by the periods of HIV diagnosis, Barcelona, Spain, 1982–2020 (n = 5,377)

^a^ At time of data extraction in December 2020.

At the time of data extraction, women of 50 years or older accounted for 68% (564/828) of all WLWH in active follow-up, compared with 42% (1,928/4,549) of men in the same age group **(** **Figure 3** **)**.

Only one individual among the 5,377 in active follow up depicted, was under 20 years old, so the < 20-year-age category is not visible on the graph.

The main mode of HIV acquisition for women diagnosed in the first part of the study (1982–1989), was intravenous drug injection (59%, 57/96, with three women among the 96 lacking information on mode of HIV acquisition). After that period, sexual contact was the primary mode of acquisition (70%, 192/274 in 1990‒1996; 82%, 181/222 in 1997‒2004; 88%, 88/100 in 2005‒2009; 81%, 61/75 in 2010‒2014 and 89%, 54/61 in 2015‒2020). A similar phenomenon was observed in men **(** Supplementary Figure 1 **).**

**Figure 4:**
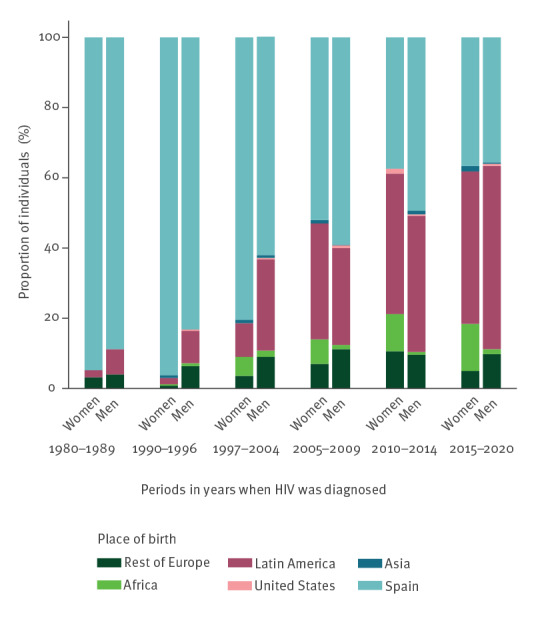
Respective proportions of men and women living with HIV according to their place of birth and period of diagnosis, Barcelona, Spain, 1982–2020 (n = 4,518)^a^

^a^ Information on place of birth was missing for 851 men and eight women.

Regarding the place of birth, a progressive increase in the percentage of foreign-born men and women, composed mainly of people originating from Latin America, has been observed since 1997. Overall, in the active cohort, men born in Latin America accounted for 34% (1,273/3,698) of the MLWH with available information on place of birth, and women born in Latin America accounted for 14% (117/820) of WLWH with such information. Men born in Latin America represented only 9% (33/388) of the active cohort before 1997. However, in the period 2015–2020, they represented 52% (466/894) of those with a new diagnosis, in contrast to 36% (318/894) for men born in Spain. The same phenomenon was observed for women in the active cohort where, in terms of HIV diagnoses, proportions of women originating from Latin America outnumbered those of women born in Spain in the last two periods of the study, with respectively 40% (30/75) vs 37% (28/75) in the 2010–2014 period, and 43% (26/60) vs 37% (22/60) in the 2015–2020 period **(** **Figure 4** **).**

### HIV viral load, CD4^+^ cell count at diagnosis, and nadir CD4^+^ cell count

As explained in the Methods section, only treatment-naïve WLWH (n = 549) and MLWH (n = 2,787) who were diagnosed with HIV after 1990 were included in the immunovirological data analyses.

**Figure 5:**
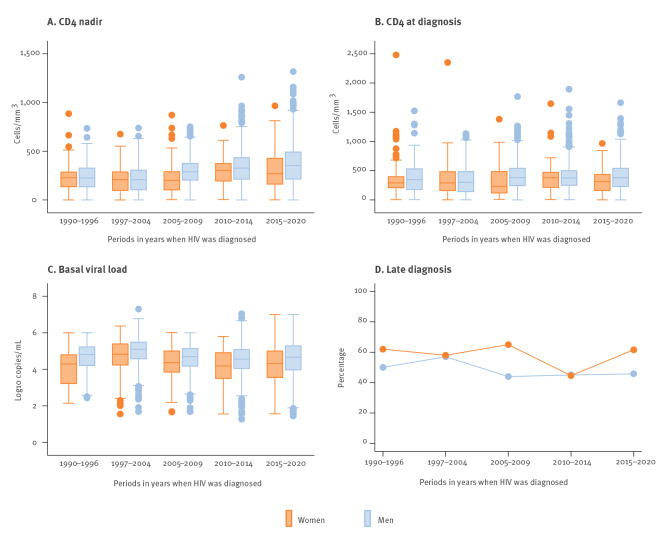
Characteristics of treatment-naïve HIV patients in terms of (A) nadir CD4^+^ cell count (cells/mm^3^) (n = 3,334)^a^, (B) CD4^+^ cell count at diagnosis (cells/mm^3^) (n = 3,277)^a^, (C) basal log viral load (n = 3,179)^a^, (D) late diagnosis (n = 3,277)^a^, Barcelona, Spain, 1990‒2020

^a^ Numbers presented concern individuals among the 3,336 between 1990 and 2020, who had data available for the parameters in question.

The viral load at diagnosis was lower in the women (median: 4.47 log_10_ copies/mL; IQR: 3.77–5.15) than in the men (median: 4.73 log_10_ copies/mL; IQR: 4.13–5.28), and this difference was statistically significant in all periods **(** **Table 1** **),** except for in the period of 2005–2009, in which, despite clinical relevance, the p value was close to being statistically significant though not (p = 0.055) **(** **Figure 5-C** **).**

Box lines represent the 25th (Q1), 50th (median), and 75th (Q3) percentiles. Adjacent lines represent the lower and upper adjacent values (i.e. lowest and highest observations that are still within the range defined as follows: Q1 − 1.5 × IQR for the lower limit; Q3 − 1.5 × IQR for the upper limit). Dots represent outliers.

The median basal CD4^+^ cell count at diagnosis for women was 289 cells/mm^3^ (IQR: 176–457), and there was no difference between the periods. The median basal CD4^+^ cell count at diagnosis in men was 365 cells/mm^3^ (IQR: 213–528). These values were significantly lower in the women than in the men in the periods 2005–2009 (p = 0.024) and 2015–2020 (p = 0.048) **(** **Table 1** and **Figure 5-B**).

The nadir CD4^+^ cell count was lower in the women (median: 225 cells/mm^3^; IQR: 114–310) than in the men (median: 289 cells/mm^3^; IQR: 169–409), and the difference was statistically significant for the same periods as those for the low basal CD4^+^ cell count **(** **Table 1** and **Figure 5-A** **).**

Overall, a higher percentage of women than men presented with a late diagnosis (CD4^+^ cells/mm^3^ < 350 at the first visit). In the last period (2015–2020), 62% of the women had a late diagnosis, whereas 46% of the men had a late diagnosis (p = 0.030) **(** **Table 1** **)**, with no differences observed between the Spanish and foreign-born women **(** **Table 2** **)**. Women who presented with a late diagnosis in the periods 1997–2004 and 2015–2020 were significantly older than those who did not, with medians of 35 years old (IQR: 30‒44) vs 31 years old (IQR: 26‒37) (p = 0.006) and 37 years old (IQR: 32–45) vs 33 years old (IQR: 25.5‒42) (p = 0.039), respectively. Except for the period 2010–2014, the percentage of late diagnosis in women remained stable at ca 60%. In contrast, there was a progressive decrease in the percentage of a late diagnosis in men after 2004, after which it remained stable at ca 45% of all new diagnoses **(** **Table 1** and **Figure 5-D** **)**.

### Antiretroviral therapy (ART) and virological failure

The analysis of the first ART was performed in only the actively-followed-treatment-naïve HIV patients who were diagnosed with HIV after 1990. The first regimen of ART changed over time according to drug approval and treatment guidelines. Of the women diagnosed in the period 2015–2020, 75% (38/51) received the first regimen based on integrase strand transfer inhibitors (INSTI), and a similar percentage of men did (73%; 479/655), which is compared with 21% (12/56) and 22% (174/777) in women and men, respectively, in the previous period (2010–2014). Changes in ART were more frequent in women than in men who were diagnosed in the first period until 2009. After that year, there were no differences in the numbers of ARTs administered between the women and men **(** Supplementary Figure 2 **).**

**Figure 6:**
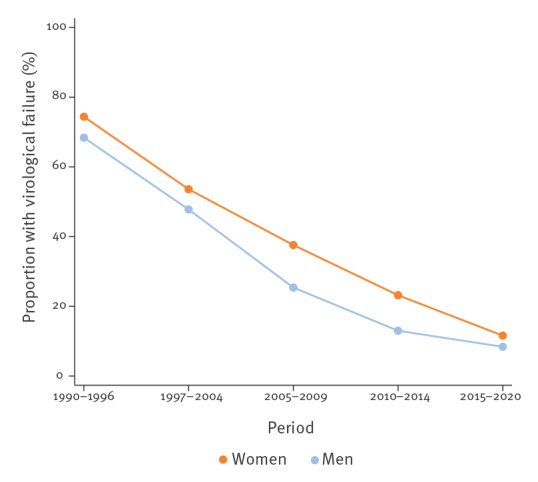
Line graph representing the proportion of individuals with virological failure among treatment-naïve HIV patients after introduction of ART and viral suppression achievement, Barcelona, Spain, 1990–2020 (n = 3,336)

Once ART was started, 99% (547/549) of treatment-naïve WLWH achieved an undetectable viral load at any point. Virological failure (defined as two consecutive viral loads of > 50 copies/mL after starting ART and achieving viral suppression) after 2005 was significantly higher in the women than in the men until December 2014 (p = 0.023 and p = 0.036 in the periods 2005–2009 and 2010–2014, respectively) **(** **Table 1** **)**, with no differences between women born in Spain and foreign-born women **(** **Table 2** **)**. However, the proportion of women with virological failure sharply decreased in the period 2015–2020, when it was not significantly different from the proportion of men with virological failure (12% vs 8%; p = 0.431) **(** **Table 1** and **Figure 6** **).**

## Discussion

Women represented 18% of all PLWH who visited our cohort at Hospital Clínic (Barcelona), which is a similar result to what has been recently published in a Spanish national cohort analysis, where women represented 16.6% of the total cohort [7].

The number of women who visited our centre and who had a diagnostic examination between 2015 and 2020 has remained low and was much lower than that of men.

When we compared women and men who are in active follow-up at our centre, we observed a similar distribution in both the current age and age at diagnosis. A younger age at diagnosis was observed in the first period of the study, probably because intravenous drug injection was the main mode of HIV acquisition in that period. When we compared age by the place of birth in women, a younger age at diagnosis was observed in the foreign-born women than in the Spanish women after 2005. This finding is relevant because of the childbearing potential of younger women and the possibility that many of them may be nulliparous at the time of diagnosis. It would then be necessary to provide adequate information and adapt initial ART in these cases [8]. Possible reasons for the younger age at diagnosis in foreign-born women (most of them being Latin American) than in Spanish women could be a higher perception of risk in this community [9] or an earlier screening in the reproductive health controls due to pregnancies at younger ages among the immigrant population in Spain, as stated in the last published report of the Spanish National Institute of Statistics [10]. If these were the causes, we would expect to see a higher rate of a late diagnosis in Spanish women; however, we did not observe any differences between the foreign-born and women born in Spain in the rate of a late diagnosis. Another possible explanation for the younger age at diagnosis of foreign-born women could be exposure to risks for HIV at an earlier age than women born in Spain, however, to the best of our knowledge, we are not aware of reports to support this hypothesis.

The introduction and constant improvement in ART has translated into a prolonged life expectancy in PLWH. HIV is now considered a chronic condition, and PLWH present distinct comorbidities according to age, in addition to possible adverse long-term effects of antiretroviral medication and the HIV infection itself. The fact that women aged > 50 years accounted for almost 70% of all women in active follow-up in our cohort raises concerns about the need to screen for pathologies that are prevalent in women at this age, such as osteoporosis, dyslipidaemia, and cardiovascular diseases, to include them in preventive strategies (i.e. breast cancer screening), in addition to addressing potential long-term adverse effects of previous or current ART.

A progressive increase in the number of HIV diagnoses in people from Latin America has been observed, which coincides with the migratory flow into Spain during the past 20 years [11]. The Spanish HIV surveillance agency reported that 33.9% of new HIV diagnoses in Spain in 2020 were in foreign-born individuals [4].

These findings are consistent with those presented in recent years by various Spanish working groups [7,12,13], and they have important clinical implications because they have been related to differences in aspects such as risk behaviours and diagnostic delays (although not demonstrated in our cohort), coinfection with other pathogens at diagnosis, presentation of disease, adherence to treatment, or treatment failure [12,14].

Sexual transmission was the main mode of HIV acquisition in both the men and women in the active cohort. Intravenous drug injection as a mode of acquisition in our cohort was mainly limited to those patients still in follow-up who were diagnosed in early years, in accordance with what has been observed in the rest of the European Union (EU), and in contrast to that observed in the Eastern part of the WHO European Region, where rates of people who inject drugs in PLWH remain high.

The percentage of individuals in our cohort with a late diagnosis was high and was significantly higher in women than in men. In addition, according to the year of diagnosis, the percentage of those with a late diagnosis has remained stable over time in the women’s cohort, being as high as 62% in the 2015–2020 period, in comparison with the progressive decrease observed in men. Our findings differ from those reported in the EU, where a decrease in the percentage of those with a late diagnosis in the past 10 years has occurred for all groups and modes of transmission [15].

Some studies performed in Spain have described the ‘migrant status’ as a risk factor for a late diagnosis of HIV [16]. However, we did not observe any differences between the Spanish- and foreign-born women. These findings are in line with those recently presented by the European Centre for Disease Prevention and Control (ECDC) and the WHO Regional Office for Europe, in which the possible risk factors and predictors for a late diagnosis among European women were analysed. No association was demonstrated between a late diagnosis and migrant status, but there was an association with age (older age at diagnosis was associated with a higher risk of a late diagnosis) and the mode of acquisition only in the Eastern part of the WHO European Region (where women who acquired HIV through drug injection were less likely to have a late diagnosis than women who acquired HIV through heterosexual transmission) [17].

Having a late diagnosis is associated with high morbidity and short-term mortality [18,19]. According to the 2020 data from the ECDC, 51% of those with a new infection were diagnosed with HIV while having a CD4^+^ cell count of less than 350 cells/mm^3^. A late diagnosis was more frequent in women, older adults, people who acquired HIV through heterosexual sex, people who inject drugs, and migrants from South and South-east Asia, sub-Saharan Africa, and central and eastern Europe. The last epidemiological surveillance bulletin for HIV and AIDS in Spain reported that the percentage of late diagnoses was 49.8% and that the percentage was higher in women than in men (54.4% vs 49.2%) [4]. These findings are similar to those observed in our cohort.

It is striking that 40 years after the onset of the HIV pandemic, the percentage of those with a late diagnosis did not decrease considerably. Although evidence suggests that the current tools of European health services (including sexual health programmes, quality of care in primary care centres, notification systems for sexual partners, and tests for patients with indicator diseases) are effective interventions that favour an early diagnosis, their coverage is still limited [15,20]. This means that further efforts towards HIV prevention across Europe are warranted to achieve universal health coverage for all and meet the Sustainable Development Goal 3 target of ending AIDS by 2030 [21].

From a virological point of view, women presented significantly lower viral loads than men at the time of entering the cohort throughout all periods. Lower viral loads at diagnosis in women have been widely described in the literature since the early years of research on HIV [22-24]. This finding has been recognised as of special importance in terms of curative strategies [25].

Women achieved viral suppression in the majority of cases, as did men. However, women experience more episodes of virological failure. Some studies performed in the United States have reported higher rates of virological failure in women in relation to ethnic and sociodemographic factors [26]. Studies in the first decade of the present century reported poorer treatment adherence in women [27], possibly related to a higher prevalence of adverse effects in women than in men [28]. Differences in ART tolerance have been reported in a few studies [29]. Adverse effects are a well-documented reason for changing ART, and actual evidence demonstrates the need for ART changes to improve the quality of life of PLWH [30]. In our study, changes in ART before 2009 were more frequent in women than in men, but men and women exhibited similar rates after that year. A Swedish study recently described that the frequency of reported side effects significantly decreased from 2011 to 2017, coinciding with a shift in ART prescriptions from efavirenz to dolutegravir [31]. Currently, as many as 62% of the women in our cohort are undergoing INSTI treatment, and episodes of virological failure have been far less frequent in our cohort since 2015, which coincides with the introduction of second-generation INSTIs. This could be explained by the better tolerance to ART exhibited by this group.

However, weight gain after the initiation of ART is a growing concern in PLWH. In a pooled analysis of eight randomised clinical trials from 2003 to 2019 by Sax et al. (2020), regimens containing INSTI were associated with greater weight gain than non-nucleoside reverse transcriptase inhibitor (NNRTI)-based or protease inhibitor (PI)-based regimens [32]. Demographic factors, such as female sex and black race/ethnicity have also been associated with greater weight gain [33,34]. This last issue requires research efforts to elucidate the causes of weight gain with these regimens that specifically affect women and to be able to offer other options that are equivalent in efficacy and tolerance.

The main strengths of this study are the large number of PLWH included, long-term follow-up, and analysis of the results by periods, which helps to consider the different epidemiological contexts throughout the HIV pandemic and its evolution in our area. However, we are aware of the limitations of this study. Some data were missing owing to its retrospective nature, and we did not include all PLWH who had visited the hospital for data accuracy reasons and only analysed patients who were in current follow-up. Finally, the fact that this was a single-centre study makes it difficult to extrapolate results to other contexts/areas. There is a high percentage of Latin American immigrants in Barcelona (cf.d with the rest of Spain); however, immigrants from other areas are lacking or underrepresented.

## Conclusions

In conclusion, the diagnosis of HIV in women in our area has decreased significantly in recent years. However, a steady and progressive increase in Latin American women with HIV has been observed, and in 2015–2020, they represent almost half of new HIV diagnoses at a younger age (i.e. < 40 years old) in women. The percentage of those with a late diagnosis remained high in our cohort in general, but the women had a higher percentage of a late diagnosis than the men. Finally, a high percentage of women aged ≥ 50 years are currently in follow-up. WLWH are still underrepresented in the scientific literature in demographic, epidemiological, pharmacological, and long-term follow-up. Determining these characteristics in particular settings is of great importance for establishing preventive and clinical interventions that are frequently not prioritised in favour of other populations with the highest risk.

## Supplementary Data

275000 Supplementary Material
