# Supplementary material for: Sex-based epidemiological and immunovirological characteristics of people living with HIV in current follow-up at a tertiary hospital: a comparative retrospective study, Catalonia, Spain, 1982 to 2020
Source: Euro Surveill. 2023 Mar 9;28(10):2200317. doi: 10.2807/1560-7917.ES.2023.28.10.2200317 (PMC9999459; doi:10.2807/1560-7917.ES.2023.28.10.2200317)
Supplement: Supplementary Material [file 2200317_SupplementaryMaterial.pdf]

This supplementary material is hosted by *Eurosurveillance* as supporting information alongside the article *'Sex-based epidemiological and immunovirological characteristics of people living with HIV in current follow-up at a tertiary hospital: a comparative retrospective study, Catalonia, Spain, 1982 to 2020'*, on behalf of the authors, who remain responsible for the accuracy and appropriateness of the content. The same standards for ethics, copyright, attributions and permissions as for the article apply. Supplements are not edited by *Eurosurveillance* and the journal is not responsible for the maintenance of any links or email addresses provided therein.

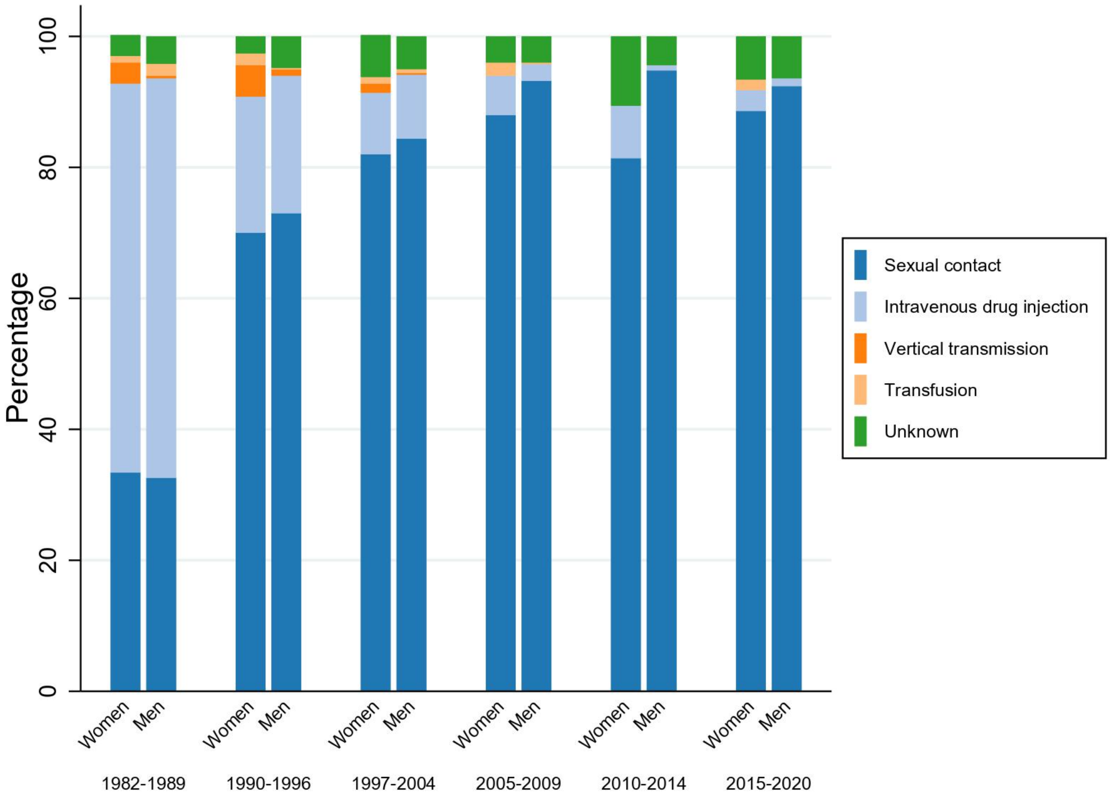

Supplementary figure 1. Mode of HIV adquisition by sex. Absolute percentages are shown in the y-axis. Year of HIV diagnosis is shown in the x-axis.

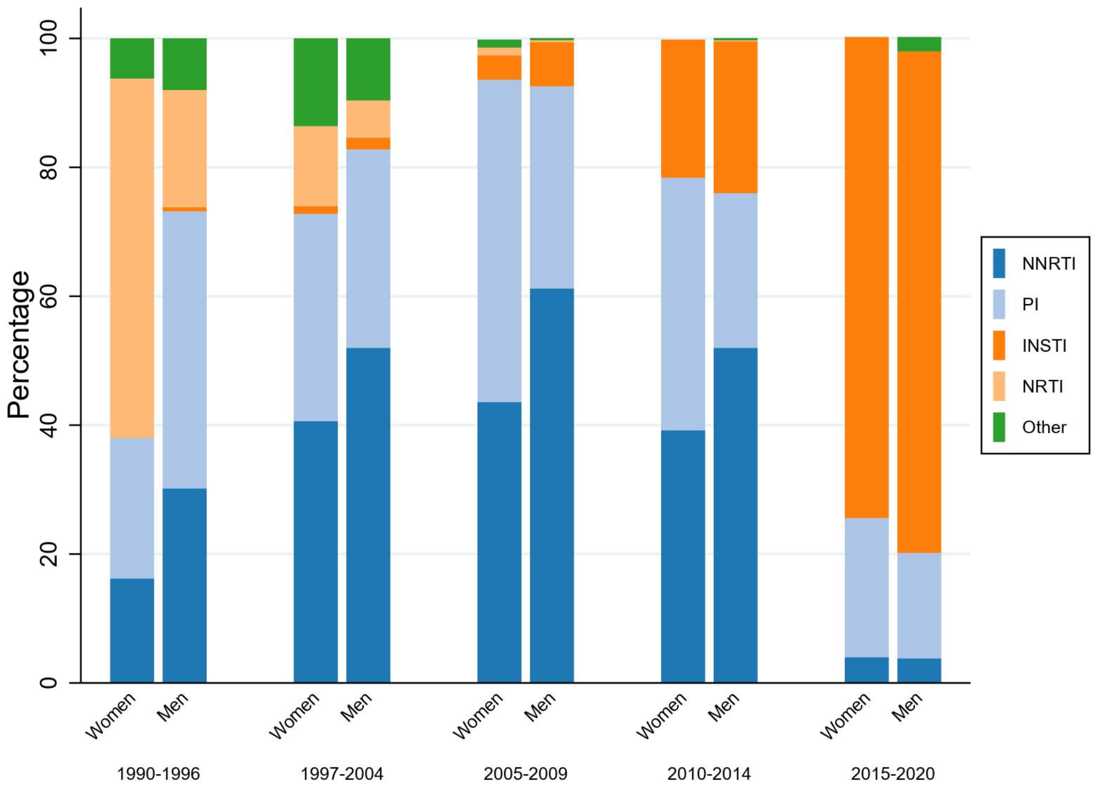

Supplementary figure 2. Type of first ART in naïve patients. Absolute percentages are shown in the y-axis. Year of HIV diagnosis is shown in the x-axis. Please note that the x-axis indicates the diagnostic period, which does not necessarily have to coincide with the start of ART (especially in people diagnosed before 2015).
